# The 5Ws of Racial Equity in Research: A Framework for Applying a Racial Equity Lens Throughout the Research Process

**DOI:** 10.1089/heq.2022.0042

**Published:** 2022-12-16

**Authors:** Keisha L. Bentley-Edwards, Patrice Jordan Fleming, Irene A. Doherty, Dane R. Whicker, Sabrena Mervin-Blake, Nadine J. Barrett

**Affiliations:** ^1^ Division of General Internal Medicine, Department of Medicine, Duke University School of Medicine, Durham, North Carolina, USA.; ^2^ Samuel DuBois Cook Center on Social Equity, Duke University, Durham, North Carolina, USA.; ^3^ Center for Equity in Research, Duke Clinical and Translational Science Institute, Duke University, Durham, North Carolina, USA.; ^4^ Community Engaged Research Initiative, Duke Clinical and Translational Science Institute, Duke University, Durham, North Carolina, USA.; ^5^ Julius L. Chambers Biomedical Biotechnology Research Institute, North Carolina Central University, Durham, North Carolina, USA.; ^6^ RCMI Center for Health Disparities Research, North Carolina Central University, Durham, North Carolina, USA.; ^7^ Department of Psychiatry and Behavioral Sciences, Duke University School of Medicine, Durham, North Carolina, USA.; ^8^ Department of Family Medicine and Community Health, Duke University School of Medicine, Durham, North Carolina, USA.; ^9^ Duke Cancer Institute, Duke University, Durham, North Carolina, USA.

**Keywords:** health equity, equity in research, racial inequities, research methods

## Abstract

Ensuring equity in research is a critical step in advancing health equity. In this perspective, the authors introduce a guiding framework for advancing racial equity in research processes, environments, and among the research workforce, the 5Ws of Racial Equity in Research. Centering their discussion on the 5Ws: Who, What, When, Where, and Why, they use historical and contemporary examples of research inequities to demonstrate how these five simple questions can encourage open discussion and proactive planning for equity in research. They close with an acknowledgment of the framework's broad utility and a researcher-directed call to action.

## Introduction

The need for more equitable research environments and practices has been well established.^1–5^ Although researchers and institutions increasingly show interest in aligning their anti-racism values with this call, many remain uncertain about how to reach more equitable ideals. Such uncertainties, especially those regarding best practices for infusing racial equity into the research process, can lead to misperceptions that equity-related challenges are not easily overcome. Whether motivated by ethical obligation, bolstering scholarly rigor, or an influx in equity-centered funding opportunities, equipping researchers with tools to apply a racial equity lens at all stages of the research process is an urgent task.

**FIG. 1.**
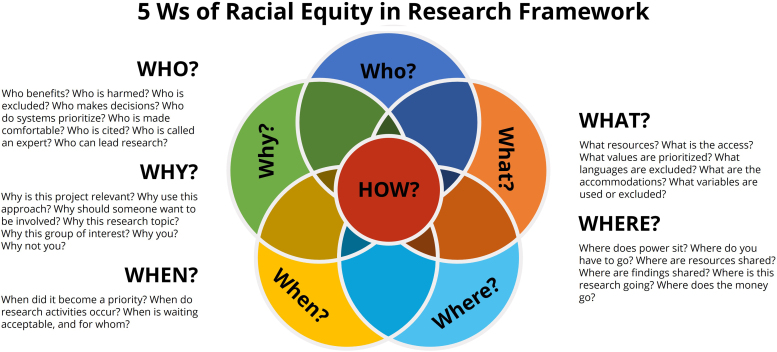
Starting questions for promoting racial equity in research. This figure provides a list of starting questions to get researchers and institutions thinking about how they can create more equitable research processes and environments.

The framework follows Aristotle's concept of deliberate inquiry in understanding processes, circumstance, and intent,^6,7^ and applies them to the necessary information gathering to conduct equitable research. As such, the 5Ws of Racial Equity in Research framework uses the *septem circumstantiae*, or Who, What, When, Where, and Why, to offer simple and relatable guidance (the how) for promoting racial equity in research processes and among the research workforce. Figure 1 provides a comprehensive, but not exhaustive, list of 5Ws questions that researchers can share with their institutions and research teams. As noted by the figure, framework questions and responses often intersect—which is a function of deliberate inquiry. This commentary pulls specific questions for deeper exploration into the 5Ws of Racial Equity in Research framework.

## Who: A Focus on People and Persons

The “who” in research often revolves around participants and recruitment. This is important when considering the over-representation of White middle-class research participants, and the underrepresentation of Black, Latinx, Indigenous, and other people of color. However, we argue that the question of “who” extends beyond participants and into all parties involved in research from conception to dissemination.

History provides critical lessons learned regarding who is included and who is excluded from research. The Tuskegee Study of Untreated Syphilis in the Negro Male,^8^ one of the most well-known examples of unethical medical experimentation, highlights potential for both intentional and unintentional disconnects between who is selected for research participation and who is included in decision making. Colloquially known as the Tuskegee Experiment, this study actively engaged Black professionals from Macon County, Alabama's educational and health institutions to aid in the recruitment and retention of poor Black men as participants.

Although Black nurses, physicians, and historically Black colleges and universities were involved, or at least aware of the study, none were in positions to make decisions or intercede on the study methods and practices that denied access to standards of care for syphilis to roughly 400 Black men for 40 years.^9^ In considering who is involved in research, engaging with community cannot be limited to recruitment assistance, or even cursory feedback on study materials. Ultimately, who is included as coinvestigators, co-awardees, coauthors, and key personnel represent whose input and interests are prioritized. When decisions are made, research teams should be asking, “Who is in the room? Who is not in the room? and Who should be in the room?”

## What: A Focus on Resources and Access

The “what” in research refers to the circumstances and extent of research activities. As such, research budgets are often in conflict with research objectives and activities. This leads to the question of whether or not resources are deemed relevant, affordable, or valuable. With budget cuts and reallocations, resources needed to achieve racial equity, such as support for community engagement, can regrettably move from a necessity to contingency status. What resources and accommodations are needed such that engagement comes at minimal burden and maximum benefit to participants, community health workers, and community advisory board members? What resources could prevent the unnecessary exclusion of racially and ethnically minoritized groups?

A recent study found a significant portion of U.S. clinical trials, especially federally funded trials, excluded participants who were not proficient in English.^10^ Budgeting for the provision of professional language translation, transportation, childcare, family-friendly research environments, and other needs creates easier paths to research participation for all. Fundamentally, the resources prioritized in research budgets should reflect the researcher's equitable values.

## When: A Focus on Time and Waiting

Whether its project timelines, funding periods, or deadlines, research is in a constant conversation with “when.” The 5W's Framework expands how researchers think about time to contemplate when research activities are conducted (whose time is a priority?) and when equity and diversity become a primary concern.

In *It's about Time: Examining the Inequalities and Time Cost of Waiting*, Holt and Vinopal provide evidence of racial and income disparities in “time autonomy,” the freedom to dictate how one's own time is allocated.^11^ Although this report heavily focused on time spent waiting for goods and services, we argue that time autonomy, and more specifically waiting, is a far-reaching, yet often trivialized and overlooked research equity issue. Are research activities carried out when most convenient for participants, or the research team? How much time is spent waiting? How many steps are involved to participate?

The U.S. Bureau of Labor Statistics has long documented racial, ethnic, income, and industry-related disparities in paid time off.^12,13^ Although most investigators recognize that participants in hourly wage jobs will not get paid while doing something else, the majority of health research still occurs during normal business hours—prioritizing the time, budgets, and schedules of research teams. Even when participants share their time and expertise with researchers, there is an expectation that their commitment to science and community supersedes financial incentives or time away from other activities, including work.

Existing research has demonstrated that participants and community health workers are motivated to engage with research for a mix of reasons that include the desire to be a part of scientific breakthroughs, improving community health, *and* financial incentives.^14,15^ To be clear, research participants, community health workers, and community partners should receive financial incentives and expend time as promised by researchers—just as much as a principal investigator's money and time is respected.

Inequities in time and waiting have also impacted the research workforce. When did it become clear that one's research team lacked racial diversity? When and how are minoritized colleagues acknowledged for their scientific contributions? The latter becomes increasingly important as more prominent White researchers launch health disparities and health equity research programs; sometimes leveraging the expertise of their racially and ethnically minoritized colleagues to obtain funding and produce high-impact publications.^16^ In the United States, marginalized communities have a long history of waiting that has historically been tied to their perceived worth. As such, careful consideration of when research is conducted and when we give credit and funding becomes a strong statement of the value placed on participants, colleagues, and community partners.

## Where: A Focus on Location and Direction

Decisions regarding where to conduct research are key to promoting equitable access and ensuring the inclusion of diverse participants. Where do individuals participate in studies? Can research activities be completed from home or is long-distance travel required? If there are academic medical centers nearby, are they trusted by the community? A history of discrimination and disinvestment in Black and Brown communities has often resulted in poor-quality health care and medical distrust among those directly and indirectly impacted by this history.^17–19^ Hesitancy to engage with medical and academic communities, whether its vaccines or participating in a research study, is deeply rooted in historical context. Hospitals and clinics can be a source of repeated trauma that is unrelated to one's medical history.

Rather, the trauma can result from negative interactions with health care providers and systems. Consequently, the implications of where we decide to conduct research extends beyond logistics and convenience. Authentic engagement includes investment in community-based facilities and businesses by contracting or having research activities in these locations rather than clinical settings whenever possible. Where research occurs and where decisions are made indicate the degree to which researchers are seeking enduring partnerships that extend beyond a funding cycle or racial reckoning. Learning from community experts occurs through true engagement, and informs where, as in what direction, current and future research should go and grow.

## Why: A Focus on Cause, Reason, and Purpose

In research, “why” speaks to intentionality in multiple ways. Utilizing a racial equity lens requires purposeful inquiry and may be the most challenging of the 5Ws questions for researchers and institutions. In the grant writing process, researchers are required to establish their intentionality through specific aims and study design. The 5Ws framework pushes researchers to deliberately evaluate their study design and ask themselves, “Why take this research approach? Is this approach rooted in racial deficit models or race norming? Why not try something else?”

Why do institutions fail to prioritize and enforce equitable research practices? History provides countless examples of structural racism's influence on research topics, questions, specific aims, recruitment methods, analytical approaches, interpretation of findings, and dissemination methods.^20,21^ Although several scholars have offered thought-provoking contributions that challenge the acceptance of these historical influences,^22–24^ adoption of anti-racist and anti-biased research methodologies remains slow. When thinking about racism and racial equity in research, there is often a focus on individual researchers and their actions. However, to answer the question of why researchers fail to become knowledgeable about, accept, and adopt such methodologies, one must also look to their institutions, who through their inaction, ultimately support maintaining an inequitable status quo.

## Discussion: Broadening the Focus

The pursuit of equity requires a proactive and strategic approach. Equity in research is a core component of scholarly rigor and ethical research practice. Researchers must be equipped to conduct their research accordingly. The 5Ws of Racial Equity in Research framework should spark increased awareness and acknowledgment of the historical contexts that cultivated contemporary inequities and infuse anti-racism, anti-bias, and ultimately equity throughout research. These contexts appear in “how” research teams are assembled, community wisdom and partnerships are engaged, research questions are selected, study designs are developed, analyses are conducted, results are interpreted, and research findings are shared. Although specifically developed to address structural, institutional, and interpersonal racism in research, the framework has broad utility and its application to other disciplines, settings, and marginalized groups is strongly encouraged.

The 5Ws of Racial Equity in Research Framework is not intended to be an exhaustive list of questions (Fig. 1), but rather a tool that encourages individual reflection, open discussion, and proactive planning among researchers and institutions as they strive to infuse equity in research infrastructure, policies, and environments. Acknowledging personal and communal processes, circumstances, and intent are required to advance equity and dismantle structural racism. Researchers and institutions must want racial equity enough to advocate, build coalitions with impacted communities, disrupt the status quo, and consistently do better.

In alignment with the 5Ws of Racial Equity in Research framework, researchers, as well as institutional leadership, must reflect on their methods and policies by asking themselves, “Who do I want to be in the fight to create more equitable research practices and environments? What am I willing to change personally and in my research agenda? When will I prioritize these changes? Where is my research going and is it seen by those most impacted by the work? And why is racial equity a priority to me now, and why wasn't it before?”
